# Deferoxamine but Not Dimethyloxalylglycine, L-Mimosine, or Cobalt Dichloride Can Interfere with the MTT Assay

**DOI:** 10.1155/2018/5872865

**Published:** 2018-11-18

**Authors:** Anna Sonja Müller, Klara Janjić, Gunpreet Oberoi, Manuela Pensch, Christoph Kurzmann, Andreas Moritz, Hermann Agis

**Affiliations:** ^1^Department of Conservative Dentistry and Periodontology, University Clinic of Dentistry, Medical University of Vienna, Vienna, Austria; ^2^Austrian Cluster for Tissue Regeneration, Vienna, Austria; ^3^Center for Medical Physics and Biomedical Engineering, Medical University Vienna, Vienna, Austria; ^4^Department of Oral Surgery, University Clinic of Dentistry, Medical University of Vienna, Vienna, Austria

## Abstract

Hypoxia mimetic agents (HMAs) have been shown to have a positive influence on cellular functions in a multitude of tissue regenerative strategies. Novel experimental approaches use biomaterials as carriers for controlled delivery of these HMAs. Here, the cytotoxic aspects of biocompatibility are of key relevance. The MTT assay is widely used to evaluate cytotoxicity and proliferation. Based on the implications from the proceeding research we hypothesized that specific HMAs such as deferoxamine at high concentrations can interfere with the MTT assay. Thus, the aim of this study was to test the repercussions of the HMAs dimethyloxalylglycine, deferoxamine, L-mimosine, and CoCl_2_ on the validity of the MTT assay. Murine MC3T3-E1 cells were cultured in serum-free alphaMEM and in alphaMEM supplemented with 10 % fetal bovine serum with the HMAs dimethyloxalylglycine, deferoxamine, L-mimosine, and CoCl_2_, respectively, at 3 mM-0.3 mM for 24 h (experimental groups). Cells without HMAs served as control (control groups). The same experiments were performed with medium and phosphate buffered saline (PBS) without cells. In all settings MTT solution was added to PBS-washed or unwashed culture plates for the last two hours of the incubation period. Then MTT solution was removed and dimethyl sulfoxide was added to dissolve the formazan crystals and absorption was measured. Our data show that the presence of deferoxamine can interfere with the MTT assay if not removed before the addition of MTT. This is particularly important when evaluating cell viability in setups where deferoxamine-loaded biomaterials are used.

## 1. Introduction

Application of hypoxia-based strategies is a promising approach in the field of regenerative dentistry and for the treatment of systemic ischemic and inflammatory diseases [1, 2]. It is met with great interest as reflected by the increasing number of publications in this field over the last ten years [1]. The use of hypoxia mimetic agents (HMAs) to simulate oxygen-deprived conditions is a common strategy applied in experimental settings [1]. In particular, when the healing capacity is compromised, biomaterials releasing HMAs can stimulate regeneration [3–5]. Previous research has shown that stem cells derived from from apical papilla release proangiogenic molecules when exposed to CoCl_2_, inducing hypoxic conditions which in turn enhances pulp regeneration [6]. HMAs furthermore increase intracellular HIF-1*α* levels, the regulator of cellular and developmental response to hypoxia, and thus lead to an increased VEGF production in dental pulp cells* in vitro* [7, 8]. Fracture healing in long bones and in mandibular distraction models in rodents improved when HMAs were applied for the activation of angiogenic factors during tissue repair [9–11]. Frequently used HMAs include dimethyloxalylglycine (DMOG), deferoxamine (DFO), L-mimosine (L-Mimo), and CoCl_2_ [1–3, 12, 13].

Various biomaterials are under development and evaluation for the release capacity of HMAs. These include collagen barrier membranes and bone substitute materials [12, 14–16]. The success of new scaffolds depends on the biocompatibility and release kinetics of these materials [17]. One of the first steps in testing new materials for the property of being an “intrinsically biocompatible system” includes cytotoxicity testing along with other qualities [18]. A well-established method to assess cytotoxicity and proliferation is the MTT assay which is based on the evaluation of the cellular metabolic activity as a measure of cell viability. The MTT assay is a colorimetric assay as oxidoreductase enzymes in viable cells convert the tetrazolium dye MTT (3-(4,5-dimethylthiazol-2-yl)-2,5-diphenyltetrazolium bromide) into dark blue formazan crystals. These formazan crystals are then dissolved with dimethyl sulfoxide (DMSO) causing purple coloration [19].

When evaluating the cytotoxicity of biomaterials which are loaded with HMAs and show a release of HMAs or when assessing the direct impact of HMAs it is important to choose a feasible assay. The MTT assay has been used for these purposes [3, 7, 8, 20]. When using this assay for evaluation of viability or proliferation, however, it is important to know whether the HMAs interfere with MTT assay leading to false positive or negative results. This is of importance, particularly when evaluating cells cultured on an HMA-loaded scaffold where HMAs cannot be removed for the assay. We found indications that the HMA DFO could interfere with MTT assay in previous studies [3, 8, 20]. However, currently no study on this issue is available although MTT assay has been used to measure viability of cells in response to DFO [7, 21]. We hypothesized that DMOG, L-Mimo, and CoCl_2_ do not interfere while DFO does interfere with the MTT assay. Thus, the aim of this study was to test the impact of the HMAs DMOG, DFO, L-Mimo, and CoCl_2_ on MTT assay.

## 2. Materials and Methods

### 2.1. Cell Culture and Plate Preparation

To assess the cell viability upon treatment with HMAs, MC3T3-E1 cells were seeded with alpha minimal essential medium (alphaMEM; Invitrogen Corporation, Carlsbad, CA, USA) supplemented with or without (serum free, SF) 10 % fetal calf serum (FCS; LifeTech, Vienna, Austria) and antibiotics in 96 well plates at 50,000 cells/cm^2^. The plates were incubated at 37 °C, 5 % CO_2_, and 95 % atmospheric moisture overnight. The HMAs DMOG, DFO, L-Mimo, and CoCl_2_ were then added at 3 mM, 1 mM, 0.3 mM, 0.1 mM, and 0.03 mM to cells with and without 10% FCS. The plates were again incubated at 37°C, 5% CO_2_, and 95% atmospheric moisture for 24 hours. In a separate set of experiments the cells were washed with PBS before the MTT assay was carried out. For the last 2 hours of incubation plates were subjected to the MTT assay.

To evaluate if HMAs can increase the absorbance in the MTT assay without cells, alphaMEM with and without 10% FCS was supplemented with the HMAs DMOG, DFO, L-Mimo, and CoCl_2_ at 3 mM, 1 mM, 0.3 mM, 0.1 mM, and 0.03 mM (experimental groups). Wells without HMA served as control (control groups). The plates were incubated at 37 °C, 5 % CO_2_, and 95 % atmospheric moisture for 24 hours and subjected to the MTT assay for the last 2 hours. In a separate set of experiments the wells were washed with PBS before the MTT assay was carried out. To reveal the impact of medium we performed the cell-free experiments also with PBS instead of alphaMEM. Here MTT assays were also performed with and without washing with PBS.

### 2.2. MTT Assay

The wells with and without cells were incubated with 1 mg/mL MTT (3-(4,5-dimethylthiazol-2-yl)-2,5-Diphenyltetrazolium Bromide; Sigma-Aldrich, St. Louis, MO, USA) at 37 °C for 2 h as previously described [14, 22–24]. MTT solution was discarded and 100 *μ*L dimethyl sulfoxide (DMSO) was added per well. Formazan formation was quantified via photometric evaluation of the absorbance at 550 nm using the Synergy HTX Multi-Mode Reader (BioTek, Winooski, VT, USA).

### 2.3. Statistical Analysis

Statistical analysis was performed with IBM SPSS Statistics (IBM Corporation, Armonk, NY, USA), using the Kruskal-Wallis-test and* post hoc* Mann-Whitney-test. The level of significance was set at* p *< 0.05.

## 3. Results and Discussion

**Figure 1:**
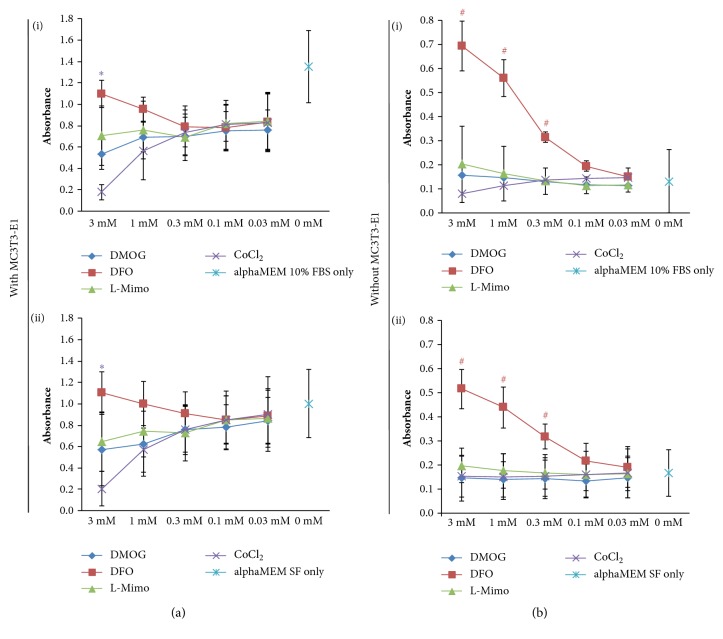
The impact of hypoxia mimetic agents measured in the MTT assay with and without the presence of MC3T3-E1 in alphaMEM with and without fetal calf serum. The MTT assay was performed in experiments with (a) and without MC3T3-E1 cells (b) with dimethyloxalylglycine (DMOG), deferoxamine (DFO), L-mimosine (L-Mimo), or CoCl_2_ in the presence of medium with and without serum (serum free, SF). Violet *∗* p < 0.05 CoCl_2_ versus the respective control (alphaMEM SF or alphaMEM with FCS); red # p < 0.05 DFO versus the respective turquoise control (alphaMEM SF only or alphaMEM with FCS only).

Utilizing the MTT assay, we first assessed the viability of MC3T3-E1 cells when exposed to four different HMAs DMOG, DFO, L-Mimo, and CoCl_2_ and compared the measured absorbance to the control, which was not exposed to any HMAs. We found that DMOG, L-Mimo, and CoCl_2_ did not substantially impair viability of MC3T3-E1 supplemented with 10% FCS at any HMA-concentration (Figure 1(a)(i)). However, CoCl_2_ induced a significant decrease at 3 mM with an absorbance of 0.180. The absorbance of the control was 1.352. For MC3T3-E1 cultured in serum-free medium (Figure 1(a)(ii)) we found similar results when treated with CoCl_2_ at 3 mM. The exposure to CoCl_2_ led to a significant decrease of viability, namely, to an absorbance of 0.205. Here the level of the control was 1.002. DMOG, L-Mimo, and CoCl_2_ did not impair cell viability significantly at the observed concentrations.

**Figure 2:**
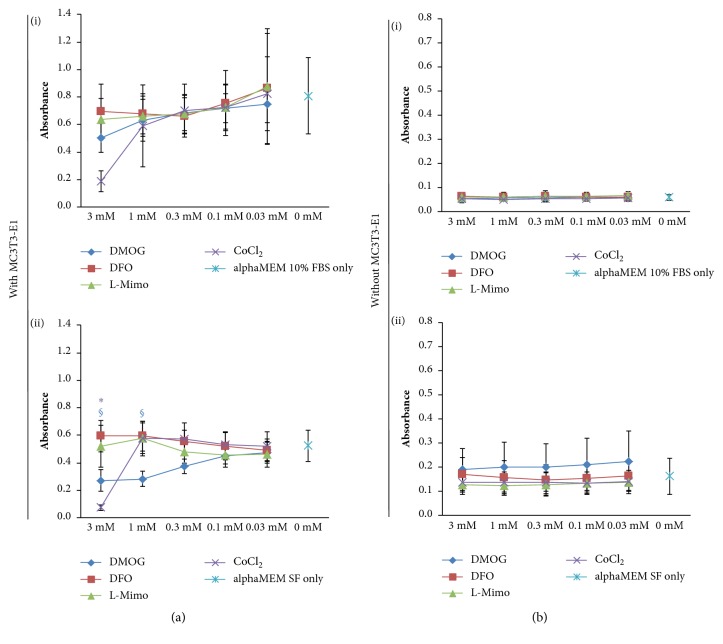
The impact of hypoxia mimetic agents after washing, measured in the MTT assay, with and without the presence of MC3T3-E1 in alphaMEM with and without serum (serum free, SF). The MTT assay was performed in experiments with (a) and without MC3T3-E1 cells (b) with dimethyloxalylglycine (DMOG), deferoxamine (DFO), L-mimosine (L-Mimo), or CoCl_2_ in the presence of medium with and without serum (serum free, SF). The plates were washed with PBS before addition of MTT. Violet *∗* p < 0.05 CoCl_2_ versus the respective control (alphaMEM SF or alphaMEM with FCS); blue § p < 0.05 DMOG versus the respective turquoise control (alphaMEM SF only or alphaMEM with FCS only).

**Figure 3:**
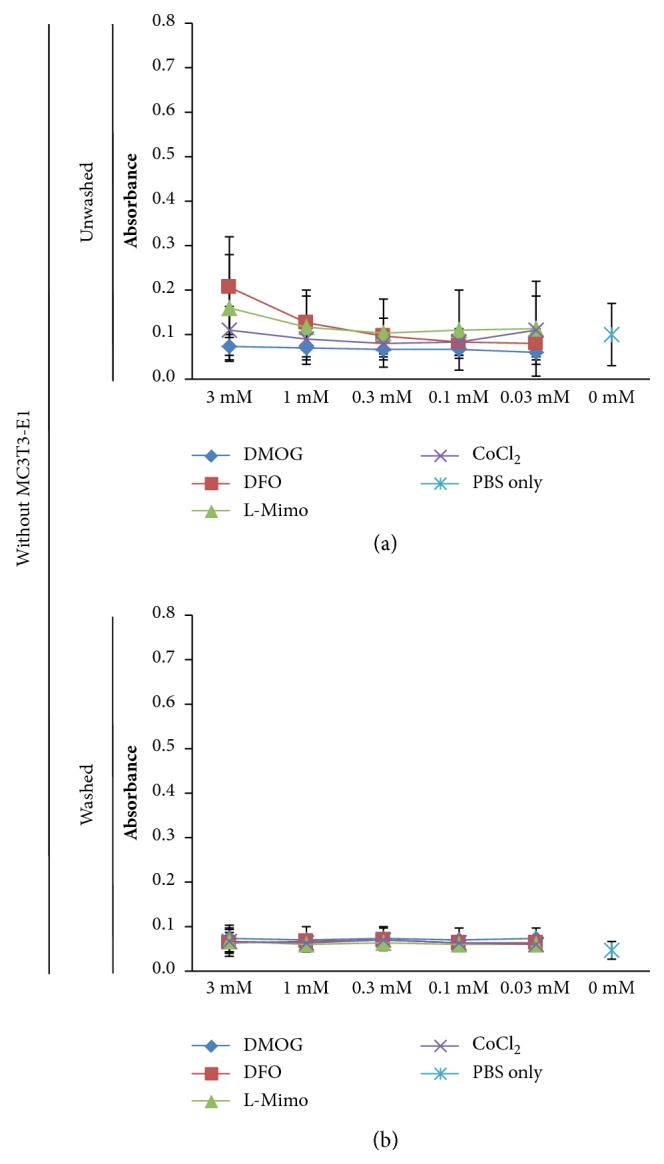
The impact of hypoxia mimetic agents, when washed or unwashed, measured in the MTT assay without the presence of MC3T3-E1 in phosphate buffered saline. The MTT assay was performed in experiments without MC3T3-E1 cells with dimethyloxalylglycine (DMOG), deferoxamine (DFO), L-mimosine (L-Mimo), or CoCl_2_ in the presence of phosphate buffered saline (PBS). The plates were either left unwashed before adding the MTT (a) or were washed with PBS before addition of MTT (b). PBS only served as control.

When performing these experiments without MC3T3-E1 cells, a pronounced increase in absorbance in the DFO treated group was observed (Figure 1(b)) while this was only observed as a nonsignificant trend in the presence of cells. The MTT assay with HMAs in the presence of alphaMEM with 10% FCS showed absorbance between 0.081 and 0.202 for DMOG, L-Mimo, and CoCl_2_ in all concentrations; thus all near to the level of the control which was 0.131. Only for DFO the absorbance values increased with the concentration reaching significant levels above 0.1 mM with a maximum at 3 mM. Here the absorbance was 0.694. The same was observed for the MTT assays carried out with HMAs in serum-free medium without cells (Figure 1(b)(ii)). Again, DFO at concentrations above 0.1 mM led to a significant increase in absorbance. The maximum absorbance for DFO was found at 3 mM with an absorbance of 0.517. The absorbance of the control was 0.167. DFO leading to such a pronounced increase in absorbance in the MTT assay in alphaMEM with and without FCS with no cells suggests that DFO reacts with a substance in the MTT assay and thus the absorbance cannot be used as a measure of cell viability in this case. We next evaluated what impact the removal of HMAs would have on the results of the MTT assay. Hence, we removed the HMAs by washing with PBS buffer before adding MTT. Here the evaluation of the optical densities showed different results. When applying MTT after washing away the HMAs the trend for an increase of absorbance by DFO was not visible, while a decrease of absorbance was observed for DMOG and CoCl_2_ at higher concentrations (Figure 2(a)). Thus, this strengthens our assumption that DFO might interfere with the MTT assay when not removed sufficiently. When performing the experiment without cells but only HMAs in alphaMEM with or without FCS including a washing step with PBS before applying the MTT assay, the absorbance levels remained similar to the levels of the control for all HMAs at all concentrations (Figure 2(b)). When the experiments were performed without MC3T3-E1 cells and no medium but only PBS, it showed no increase or decrease of absorbance compared to the control independent of adding a washing step (Figures 3(a) and 3(b)). Thus, the effect is medium dependent.

The findings of this paper are of clear relevance for researchers in the field of regenerative medicine focusing on hypoxia-based strategies. The MTT assay is a widespread tool in laboratories for cell viability testing and dose finding* in vitro*, especially in preliminary studies. Thus, potential misinterpreted results as is possible with DFO can have a major impact on follow-up studies. Especially studies on DFO-loaded materials such as bone substitutes, collagen matrices, or hydrogels need to be performed with a feasible test method [14, 21]. The impact on cell viability in the presence of high dosage of DFO might be underestimated based on the MTT results. In previous studies problems concerning possible interference of DFO with the MTT assay have been described. An increase in optical density is when human periodontal fibroblasts, human gingival fibroblasts, and dental pulp-derived cells were exposed to DFO at high concentrations (1 mM) that is why they excluded the data [3, 8]. Similar hurdles with the MTT assay were reported when murine bone marrow cultures were exposed to DFO [20]. Again, high concentrations of DFO lead to a positive reading in the MTT assay even in the absence of cells. They also excluded the MTT assay data of cells treated with DFO [20].

A limitation of our study is that we performed the experiments with only one cell line. As the conversion rate of MTT can vary in various cell types, the magnitude of the interference with the viability assay results normalized to the untreated control might also be cell type-dependent. We also used one incubation time for the MTT solution as the incubation times chosen depend on the assay manufacturer and protocol used. We incubated the MC3T3-E1 cells with MTT for 2 hours based on ISO 10993-5:2009 [25]. There, it is also stated that, depending on the cell type, different incubation times of up to 4 hours can be applied. As the same effect of the MTT assay was observed when various cells such as human dental pulp cells, human periodontal ligament cells, and murine monocytes were incubated with DFO, we do not believe that different cell types or longer incubation times would have made a major difference [3, 8, 20]. However, due to difference in the overall MTT conversion it is possible that the impact might vary with regard to the magnitude with different cell types.

Based on the results reported above we suggest that in addition to MTT other assays should be taken into consideration such as live-dead staining, resazurin based toxicity assays or bromodeoxyuridine (BrdU) incorporation assays to verify the results of the MTT assay when evaluating DFO-loaded scaffolds [21].

Future research should clarify the involved mechanism of the interference of DFO with the MTT assay.

## 4. Conclusion

The HMA DFO can interfere with the MTT assay leading to increased absorbance levels when not removed sufficiently. The absorbance increases with the DFO concentration suggesting that the impact of DFO-loaded biomaterials on cell viability can be underestimated when DFO cannot be removed before the assay.

## Data Availability

The data used to support the findings of this study are available from the corresponding author upon reasonable request.
